# A Psychometric Platform to Collect Somatosensory Sensations for Neuroprosthetic Use

**DOI:** 10.3389/fmedt.2021.619280

**Published:** 2021-03-09

**Authors:** Giacomo Valle, Francesco Iberite, Ivo Strauss, Edoardo D'Anna, Giuseppe Granata, Riccardo Di Iorio, Thomas Stieglitz, Stanisa Raspopovic, Francesco M. Petrini, Paolo M. Rossini, Silvestro Micera

**Affiliations:** ^1^The BioRobotics Institute, Scuola Superiore Sant'Anna, Pisa, Italy; ^2^Department of Health Sciences and Technology, Institute for Robotics and Intelligent Systems, ETH Zürich, Zurich, Switzerland; ^3^Bertarelli Foundation Chair in Translational Neuroengineering, Centre for Neuroprosthetics, School of Engineering, École Polytechnique Fédérale de Lausanne (EPFL), Institute of Bioengineering, Lausanne, Switzerland; ^4^Istituto di Ricovero e Cura a Carattere Scientifico (IRCCS)-Policlinic A. Gemelli Foundation, Institute of Neurology, Catholic University of the Sacred Heart, Rome, Italy; ^5^Laboratory for Biomedical Microtechnology, Department of Microsystems Engineering–IMTEK, Bernstein Center, BrainLinks-BrainTools Cluster of Excellence, University of Freiburg, Freiburg, Germany; ^6^Department Neuroscience and Neurorehabilitation, IRCCS S. Raffaele-Pisana, Rome, Italy

**Keywords:** neuroprosthesis, neurostimulation, electrodes, sensory feedback, amputees, psychophysics, somatosensations, platform

## Abstract

Somatosensory neuroprostheses exploit invasive and non-invasive feedback technologies to restore sensorimotor functions lost to disease or trauma. These devices use electrical stimulation to communicate sensory information to the brain. A sensation characterization procedure is thus necessary to determine the appropriate stimulation parameters and to establish a clear personalized map of the sensations that can be restored. Several questionnaires have been described in the literature to collect the quality, type, location, and intensity of the evoked sensations, but there is still no standard psychometric platform. Here, we propose a new psychometric system containing previously validated questionnaires on evoked sensations, which can be applied to any kind of somatosensory neuroprosthesis. The platform collects stimulation parameters used to elicit sensations and records subjects' percepts in terms of sensation location, type, quality, perceptual threshold, and intensity. It further collects data using standardized assessment questionnaires and scales, performs measurements over time, and collects phantom limb pain syndrome data. The psychometric platform is user-friendly and provides clinicians with all the information needed to assess the sensory feedback. The psychometric platform was validated with three trans-radial amputees. The platform was used to assess intraneural sensory feedback provided through implanted peripheral nerve interfaces. The proposed platform could act as a new standardized assessment toolbox to homogenize the reporting of results obtained with different technologies in the field of somatosensory neuroprosthetics.

## Introduction

**Figure 1:**
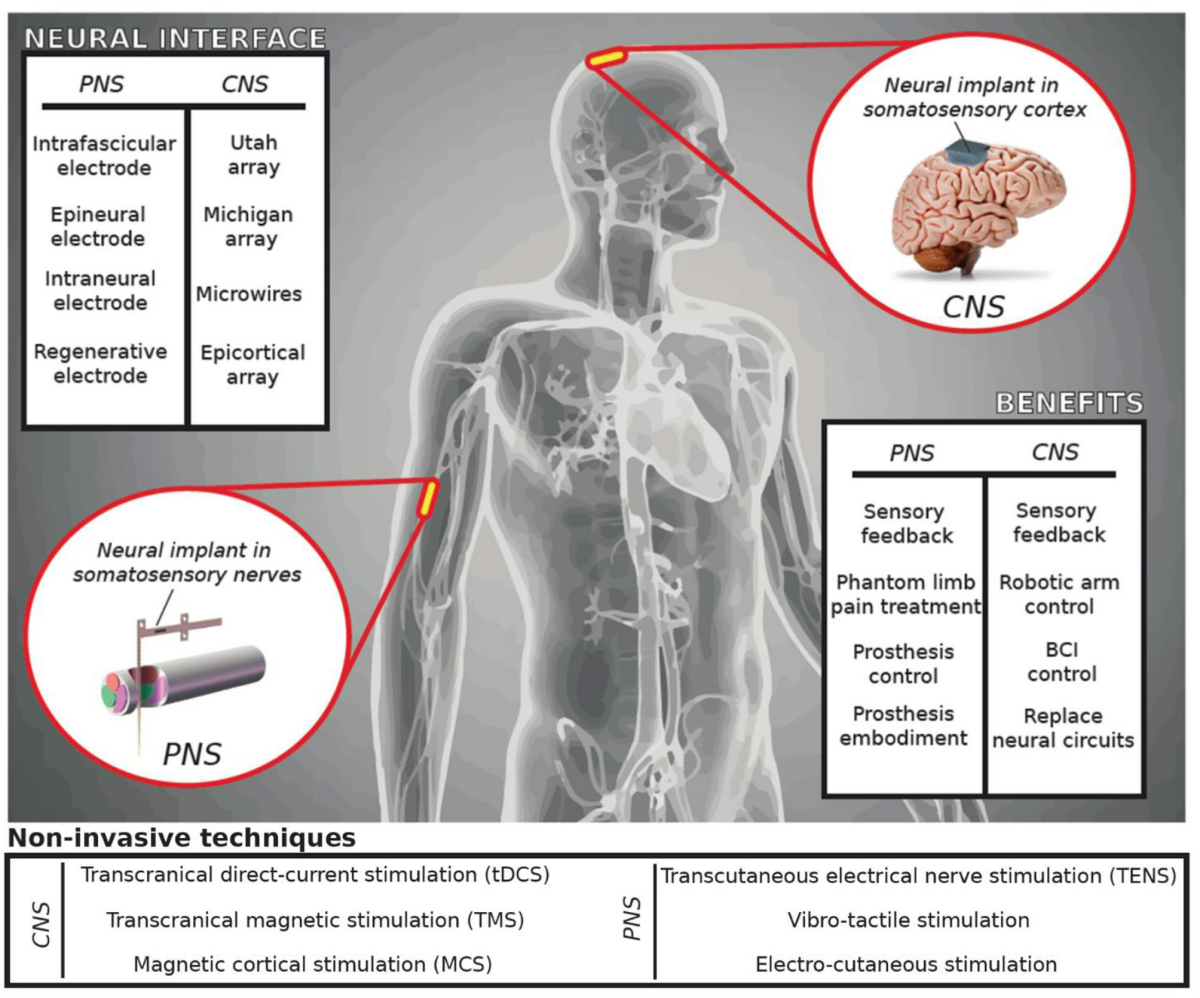
Neuroprosthetic applications. Neurotechnologies for restoring somatosensations have been developed for peripheral (PNS) or central nervous systems (CNS). The stimulation technique used to restore sensory feedback can be invasive (surgically implanted and in intimate contact with the nervous tissue) or non-invasive (applied on the skin surface). Delivering a stimulation to the brain or peripheral nerves provides benefits such as the control of robotics, smart prosthetics, or other assistive technologies.

Somatosensory neuroprostheses are highly innovative devices (1). Several research groups have investigated the ability to restore sensory feedback in patients with upper or lower limb amputation, tetraplegia, or paraplegia using invasive (2–14) and non-invasive (15–19) interfaces with the peripheral (PNS) and the central nervous systems (CNS) (Figure 1). The main aim of these technologies is to elicit somatotopic-referred sensations emanating from the affected limb, creating a personalized map of the these sensations which could be used as a sensory feedback aimed at improving the patients' quality of life (20, 21). All these approaches use neural stimulation to evoke sensations stemming from contact with sensory peripheral nerves or the neural interfaces are placed directly on the somatosensory cortex.

The external stimulation of the residual (still functional proximal to the lesion) nervous structures guarantees to evoke an artificial sensation that can also be controlled by modulating the stimulation parameters (22–24).

**Figure 2:**
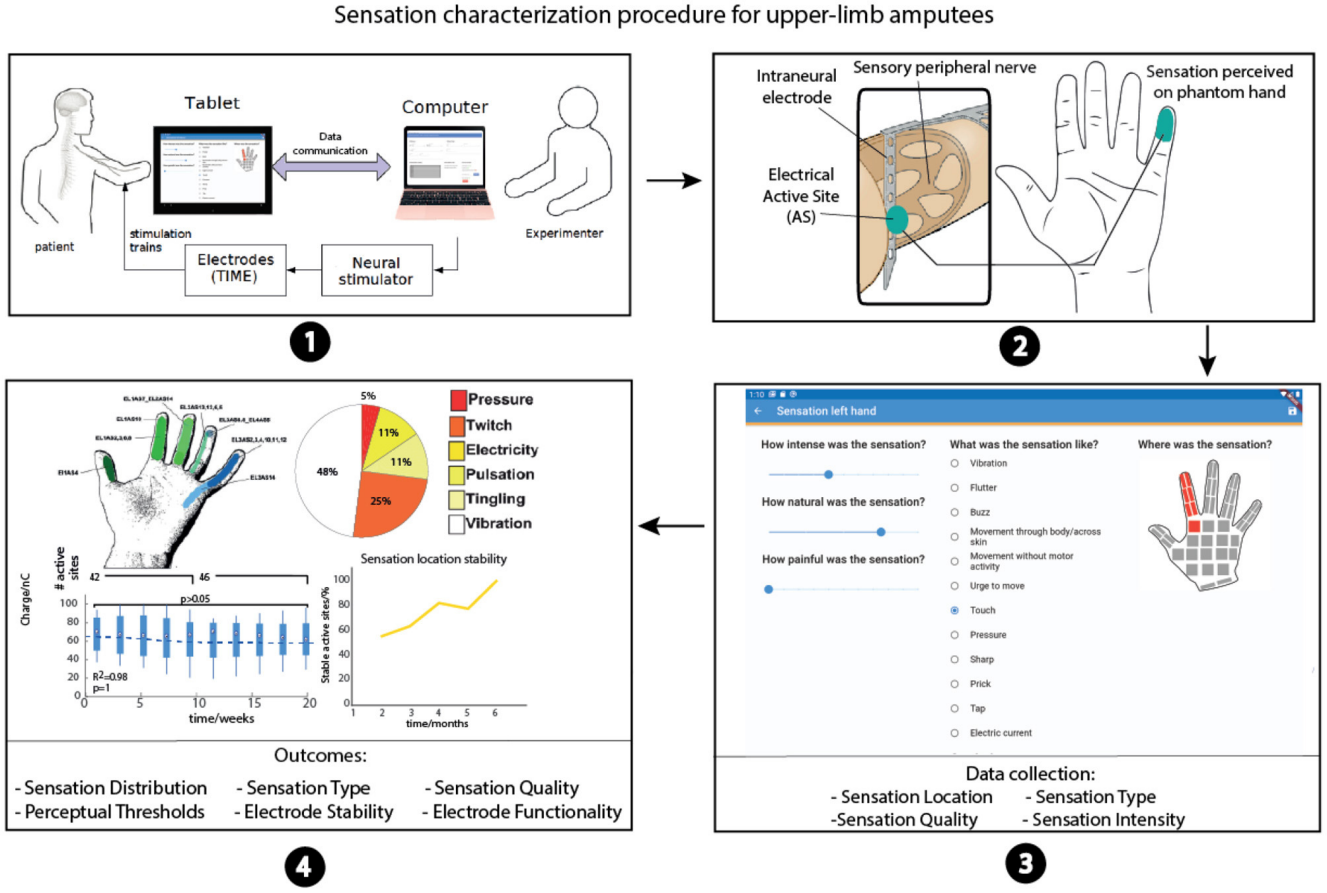
Sensation characterization procedure. (1) Stimulation parameters are selected. The stimulation trains are delivered using the neurostimulator; the software also sends control commands to the Easy Quest app. (2) Patient perceives a stimulation-evoked sensation on the phantom hand thanks to the neural implant. (3) Easy Quest app in ODF mode is used to report the sensations. (4) Experimenters collect all sensation characterization outcomes and import them in MATLAB or Excel to plot the results.

Since there is intersubject variability due to the different nerve structures, implantation levels, and innervation (25–27), together with the subjective perception of the elicited sensations, a “sensation characterization” procedure is necessary to obtain a uniform sensation mapping (Figure 2). The goal of this procedure is to collect all the stimulation parameters corresponding to the evoked sensations characterized by the intensity, quality, location, and type in order to have a personalized sensation map. The mapping phase is crucial to implement an effective real-time assistive system, e.g., bidirectional hand or leg prostheses, eliciting homologous referred sensations emanating from the phantom limb (somatotopic) for therapeutic or functional purposes. In fact, the personalized sensation map is often translated into a robotic arm or hand in order to elicit sensations during object manipulation tasks aimed at increasing patient motor control performance (4, 6, 28, 29). When the patient is controlling a robotic arm, and touches a surface with the second robotic digit, the sensation perceived should be in the same location (index), with the safety and exact intensity (mapped with the pressure force of the robotic finger) and the type should be in line with finger pressure (i.e., no electricity or warmness). The personalized sensation map should thus be as detailed as possible.

In addition, to provide a rich and reliable artificial sensory feedback to the patient, it is necessary to accurately test multiple sets of stimulation parameters and re-test them even over multiple days during long-term applications (14, 30–33). This characterization procedure is fundamental to re-create an artificial sensory feedback that could be effectively exploitable in neuroprosthetic applications by the users. To this aim, it is crucial to collect the data in an effective and efficient way using a platform in which the information are exhaustive and standardized. This would guarantee an easy comparison of the results with different technology or algorithm.

Several psychometric questionnaires exist regarding the quality and type of the sensations evoked (15, 34–36). However, they do not appear to be easy-to-use or fast for recording and integrating all the properties of the elicited sensations with detailed standard questionnaires and which could be used for several types of sensory feedback.

The psychometric platform presented in this study provides a uniform way of characterizing and quantifying the artificial sensory feedback systems used for invasive and non-invasive, peripheral, and central sensory feedback, in order to efficiently compare, optimize, and evaluate all the different approaches even over time. Our platform records the stimulation parameters, quality, type, intensity, and location of the evoked sensations. All the sensation data are collected from questionnaires already presented in the literature.

The platform also provides a user-friendly graphical user interface with a touch screen for the patient's answers that not only enables the patient to describe the percept in detail but also provides clinicians with all the main information on the evoked sensation. The platform accepts new questionnaire definitions as text and is easy to understand and implement. This means that researchers can add new questionnaires, such as phantom limb pain (PLP) (37, 38), in order to collect information on new treatments.

This psychometric platform was tested on three trans-radial amputees who had four intrafascicular electrodes (39) implanted in their median and ulnar nerves for 6 months each. The patients responded using the psychometric platform when they received electrical stimulation by the electrical contacts of the neural interfaces. The software was used by clinicians and engineers to collect the data. This has proven to be more convenient than writing down hundreds of answers in weekly trials over 18 months.

In this study, we describe the usability of this new platform. We believe that our new psychometric platform will facilitate and unify the characterization of percepts and the comparison of the effects when applying different neural stimulation techniques or using different devices.

## Methods

### Software Platform

The psychometric platform is made up of a mobile application for compiling questionnaires (which we have called Easy Quest), two desktop tools (Easy Quest Create and Easy Quest Evaluate), and a desktop application to control the neurostimulator.

The Easy Quest mobile app is described in depth in the following sections.

Easy Quest Create shows a simple graphical user interface in which the experimenter can create a list of questions from a set of predefined types. The content can be customized.

Easy Quest Evaluate is devised for the rapid evaluation of a set of answers; the software reads the archive file exported by Easy Quest and exports a comma-separated values (CSV) file. The choice of CSV format of the results makes further analyses easier, as it is compatible with MATLAB (MathWorks, Inc., Natick, Massachusetts, United States) and Microsoft Excel (Microsoft Corporation, Redmond, Washington, United States).

The desktop application for the actual neurostimulation is not described here, because its design is strongly dependent on the type of experiment and neurostimulation device (communication protocols, stimulator commands, and architecture); however it is mentioned as part of the experimental setup.

### Somatosensory Questionnaires

Somatosensory descriptors were selected from the literature and clinical settings also including questionnaires that have already been used in neuroprosthetic studies. Several options describing the type, quality, intensity, and the location are presented in order to characterize the somatosensory percepts being evoked during the stimulation. To describe the quality of sensations, we used a scale presented by Lenz et al. (34) and used also by Valle et al. (9). For the sensation type, we adapted the questionnaire proposed by Kim et al. (36) based on our experience with several upper limb patients stimulated with invasive (4, 9, 23, 24, 29, 31, 40–47) and non-invasive technologies (17, 48). We also considered other studies on sensations elicited using peripheral (5, 10, 14, 22, 49–55) or central (6, 8, 13) neural stimulation approaches. For the intensity, we used a Visual Analog Scale (VAS) (37) already presented by Tan et al. (56). Lastly, the perceived sensation locations were shown directly on a schematic representation of the human hand. It is further possible to select the feet, arms, or legs (11, 12) with several possible spots (Figure 2). In this way, the patient can accurately indicate the affected areas.

We added several questionnaires in order to collect information on PLP: VAS (37) and neuropathic pain symptom inventory (NPSI) (38). It is also possible to add or modify the existing questionnaires in order to adapt the platform to the needs and specifications of the clinical trial.

### Use Cases

**Figure 3:**
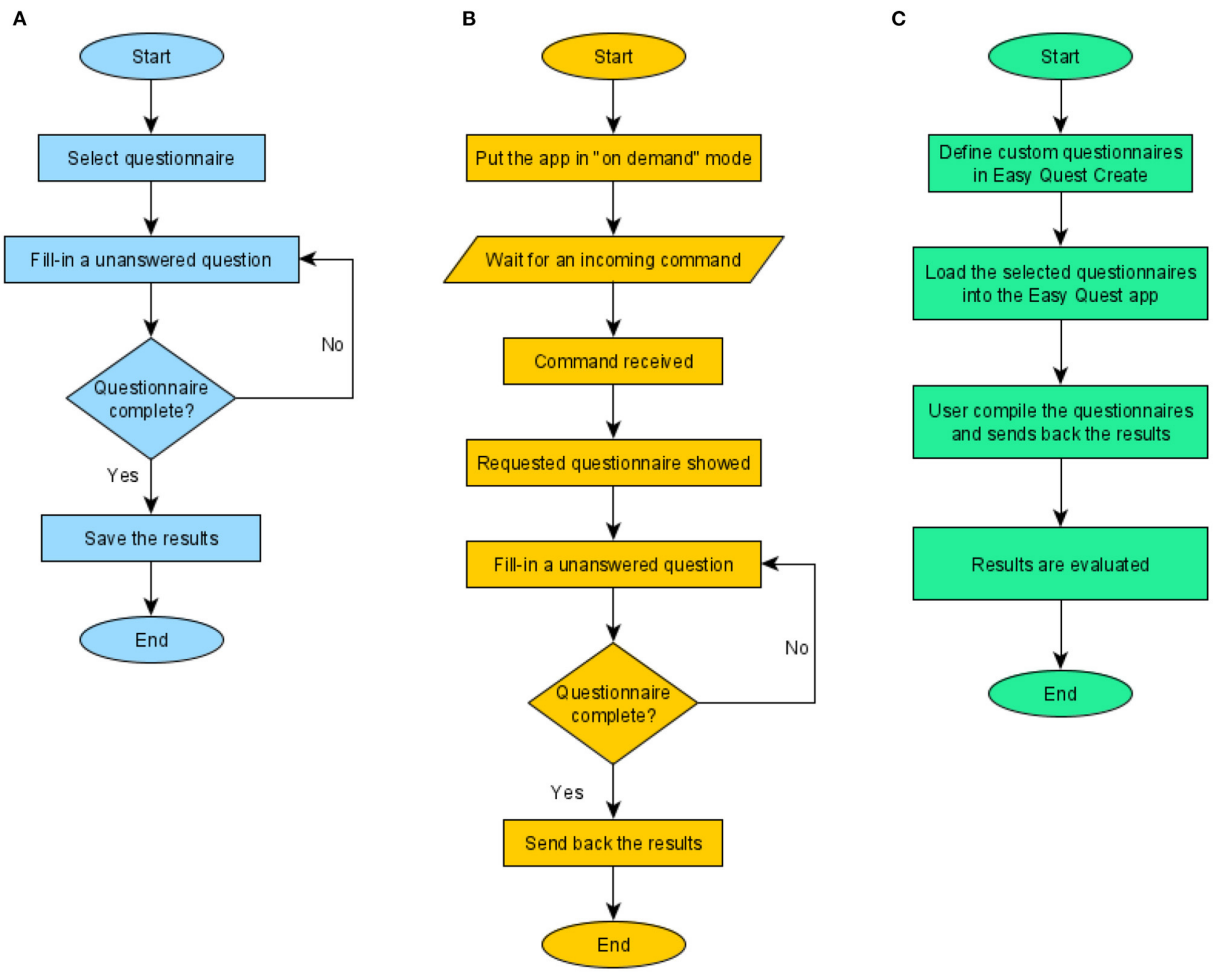
Use cases. The three main features of the psychometric platform: the first two are implemented by the mobile app and the last by the whole system. **(A)** Defined as Local Fill-in (LF), where the users compile a questionnaire and the answers are stored in the device. **(B)** On demand fill-in (ODF), in this case, the app waits for an external command from a controller app containing information on the questionnaire to be shown; the fill-in procedure is the same but nothing is stored within the device, instead results are sent back to the controller. **(C)** The procedure seen from the experimenter's point of view, here, the role of the other software programs of the platform (Easy Quest Create, Easy Quest Evaluate) is explained.

Two main use cases for the app were identified (Figure 3). In the first, the user fills in a questionnaire and saves the results on the device, defined as the “local fill-in” (LF). In the second, an external software prompts the app to show a questionnaire and to send back the results, defined as “on demand fill-in” (ODF). The two cases (Figures 3A,B) involve the same procedure in the part where the user is asked to fill in the answers.

The main difference, besides the location where the results are stored, is how the procedure starts: in the first case, the user choses a questionnaire by selecting it from the main menu, in the second, the app waits for an external command, usually from the network, instructing the software to show a specific questionnaire.

The application can set recurrent reminders for specific questionnaires, enabling the experimenter to plan the follow-up for home use by the patient, and these reminders prompt the user to fill in the questions in LF mode.

A third use case (Figure 3C) explains the workflow from the perspective of the experimenter, who uses the companion software to define new questionnaires at the beginning of the experimentation and to display the results at the end.

An explanatory example of the platform is displayed in Supplementary Video 1.

### Software Architecture

The software was developed in Dart, an object-oriented programming language developed by Google in order to address server-side, web, and mobile platforms. The mobile SDK, Flutter, compiles the code in fast native apps for Android and iOS devices.

The app is developed following the Model View Controller (MVC) pattern, and a simple Object-Relational Mapping (ORM) is implemented to store the models in an SQLite database in the device's memory. The ORM is accessed through classes which show APIs where serialized objects can be stored and retrieved.

To implement the ODF, a simple Hypertext Transfer Protocol (HTTP) server runs in the background thus the app can, when requested, wait for remote commands from the network. While doing so, the app shows a numerical code, which must be notified to the experimenter to secure the remote connection.

An interface with the mail app is used to send the completed questionnaires as a CSV file by e-mail.

Another provider class parses the questionnaires defined in JavaScript Object Notation (JSON) format, making it possible to create and add new questionnaires to working devices, without code interventions and recompiling the whole app. The import service can parse a compressed file containing a set of questionnaires and also a collection of images referred to in the questions. There are five questions accepted by the parser: (1) open, which prompts the user for a string (2); radio, which asks the user to choose one option from a set (3); multiple choice; (4) slider, where users have to select a number or a label with a slider; and (5) image touch, where the user selects a set of touchable areas displayed on top of a given background image.

The app enables multiple users to access the same device while keeping the results separate.

**Figure 4:**
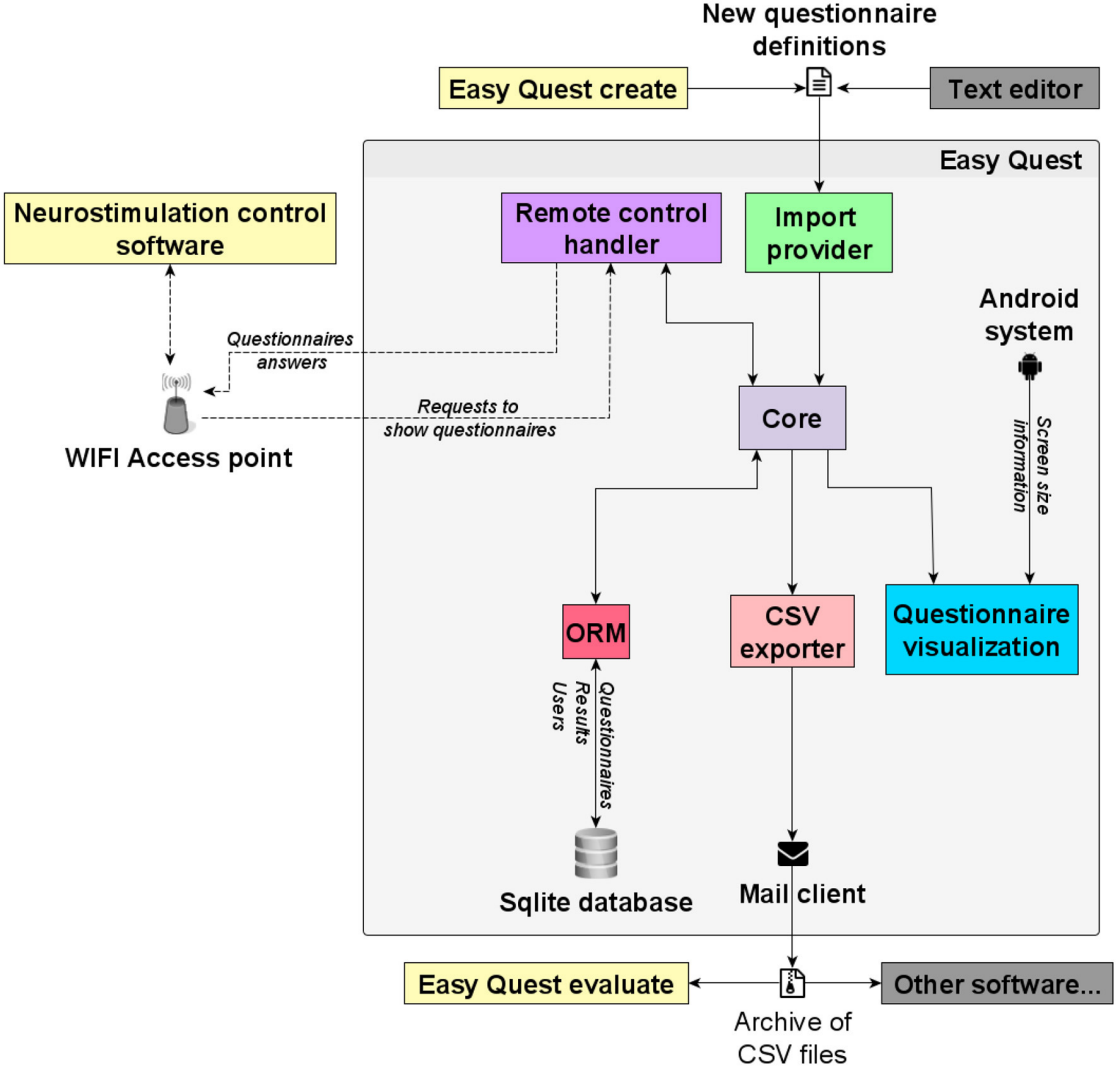
Software architecture. The main components of the platform depicted as squares, external services are shown with an icon and communication with arrows, some show a label with examples of the information flowing through. A gray shadow surrounds the software modules of the mobile app (Easy Quest).

The system architecture is shown in Figure 4, along with the external software highlighting its relationship with the app modules.

The app UI/UX is designed in accordance with Material, an open source system of guidelines developed by Google. The view layer written for the app exploits all the available space, presenting the questionnaire as a list of questions on small devices and as a grid on larger screens.

### Quality and Usability Assessment

**Figure 5:**
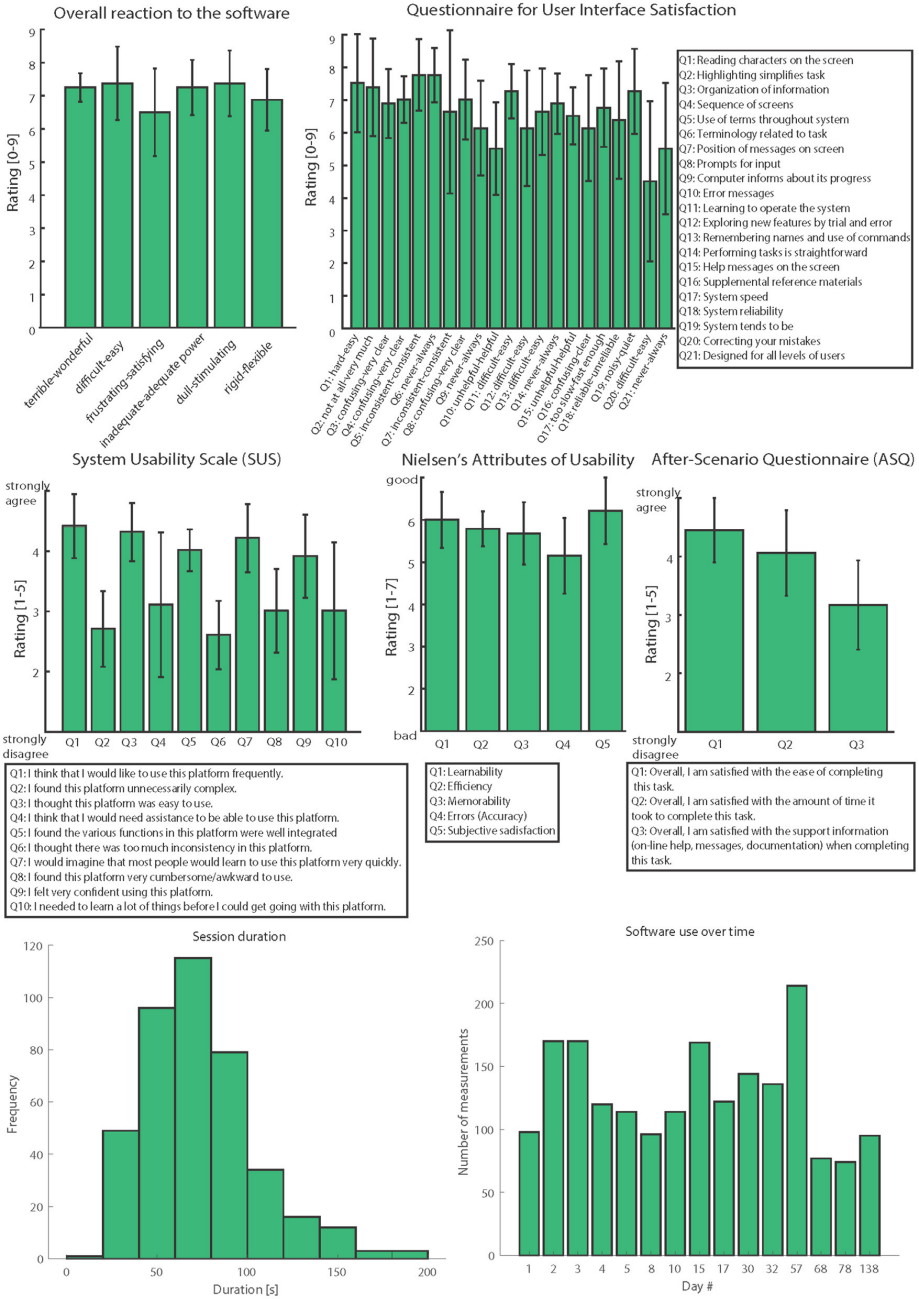
Usability assessment. All the usability scales are reported: Overall reaction to the software, QUIS, SUS, NAU, and ASQ. Three clinicians, six engineers, and three patients evaluated the psychophysical platform (*N* = 12). The data in the figure are represented as means ± standard deviations. The last row resumes usage metrics.

During the clinical trial, we collected feedback information from patients, clinicians, and engineers who used the platform presented in this study in three clinical trials (*N* = 12). The investigations regarded the development and assessment of bidirectional hand prostheses for upper limb amputees with a neural sensory feedback delivered by implantable electrodes (9, 23, 31, 41, 42). After 6 months of use, we asked participants to answer different quality and usability questions using: questionnaires for user interface satisfaction (QUIS) (57), system usability scales (SUS) (58, 59), Nielsen's attributes of usability (NAU) (60), and after-scenario questionnaires (ASQ) (61). We collected and analyzed all the information using validated and standardized questionnaires (Figure 5).

### Data Storage

Alongside the answers to the psychometric questionnaires, a MATLAB script running in experimenter's computer stored date and time for further analysis about the use of the platform. All the relevant measurements are stored as.mat files, as all the data processing have been performed inside the MATLAB software.

## Results

### Somatosensory Questionnaires for Sensation Characterization

To efficiently characterize the sensations emanating from (invasive or non-invasive) electrical (central or peripheral) stimulation, a user-friendly platform is needed with a set of somatosensory-related questionnaires. This helps to reduce the long time required to collect all the electrically evoked sensation data.

To assess the properties of the sensations being evoked by stimulating peripheral nerves using a neural interface in trans-radial amputees, we used the psychometric platform presented here. We performed a procedure called “sensation characterization” with all the patients involved in the clinical investigation (Figure 2). For each electrically active site used to stimulate the nerve, the neural stimulation was delivered, and the patient was asked to report the sensations he/she felt. This mapping phase enabled us to identify the sensation properties for all the stimulation channels of the implanted electrodes by varying the stimulation parameters and building a personalized map of the sensations. The stimulation parameters varied in terms of frequency (1–1,000 Hz), pulse-width (1–120 μs), and amplitude (1–1,000 μA), as well as stimulation train duration (discrete or continuous). We collected the sensation intensity, quality, type, and location of the patient's perceived sensations.

The intensity was used to find the perceptual thresholds for each stimulation channel (4, 23, 24, 31), together with the range of stimulation (between threshold and below pain level). Using a VAS scale in the range from 0 to 10 also enables us to identify perceptual magnitude levels (5, 23, 32, 52, 62).

The quality of the sensory feedback was assessed in order to test different stimulation strategies and approaches (9, 23), since this quality is considered to be an important factor for prosthesis acceptance (53, 63). To quantify the perception quality and naturalness, we used a scale (34) from 1 (totally unnatural) to 5 (totally natural).

The type of sensation was collected in order to understand the type of fibers being recruited during the stimulation and to identify the best channels for restoring homologous sensations while using the bidirectional prosthesis. We used 20 descriptors (Table 1) considering all the important aspects. In this platform, the patient could also report a new sensation or add comments in an empty text box when a correct descriptor for the elicited sensation was lacking.

**Table 1:** List of sensory descriptors.

Vibration
Flutter
Buzz
Movement through body/across skin
Movement without motor activity
Urge to move
Touch
Pressure
Sharp
Prick
Tap
Electric current
Shock
Pulsing
Tickle
Itch
Tingle
Numb
Warm
Cool

*Here, the chosen descriptors are shown, the user can also enter free text when the other options are insufficient*.

The sensation location was reported using a picture of the limb of interest (foot, arm, leg, or hand) with several highlighted spots (20 for foot, 24 for leg, 48 for arm, and 45 for hand) (Figure 2). The zones with a higher density of receptors had more selectable spots. This information is useful to understand the electrode stimulation selectivity (analyzing the spreading of the zone) and the layout of the fibers inside the nerve. In addition while the bidirectional prosthesis was being used, the location map was needed to stimulate the correct active sites eliciting the somatotopic sensation during the prosthesis hand/finger contact with objects (4).

Finally, several questions can be used to assess phantom limb pain levels before and after a pain treatment with electrical stimulation (31). We decided to use two different questionnaires (VAS and NPSI) to characterize the location, quality, and intensity of the pain (11, 31).

### Software Usability

The usability testing of the app was performed on an Android phone (a Nexus 6p), designed by Huawei and running Android 8.

The app loading time is <2 s, needing only the time to open the local database, and after the login screen, the user can access all the main functions in no more than two taps.

The home page shows a list of all the available questionnaires, the user can tap on each one to see the questions and fill in the answers, which are stored in the internal database.

From the lateral menu (drawer), the ODF mode can be accessed in only one tap, after which the app will wait for a network command containing the identifier of the questionnaire to be shown.

Minimal user interaction is needed to complete a questionnaire, usually all the questions need just one tap, except for the multiple choice and clickable area ones. The average time to fill in a sensation characterization questionnaire is 10 s.

The export page lets the user write all the stored data in a CSV archive file and opens the default mail to send to the experimenters for further analysis, facilitating and speeding up the data-gathering phase.

A specific section of the app lets the user choose which questionnaire should be visible in the home page, personalizing the user interface for a specific use.

Other pages are designed for secondary tasks, such as previewing stored answers and editing settings.

### Psychometric System Validation

In order to assess the usability and quality of this novel psychometric platform to collect somatosensory percepts, several questionnaires were filled in by different kinds of users. Three patients, six engineers, and three clinicians evaluated the system by answering four questionnaires after using the platform in clinical applications (Figure 5). Analyzing the results, the overall reactions to the system were very positive. The average score was 7.1 ± 0.3. Considering the user interface satisfaction (QUIS), the rating achieved was 6.6 ± 0.8. In both these questionnaires, the maximum achievable score was 9.

In the SUS (range 1–5), Q1-Q3-Q5-Q7-Q9 scored 4 ± 0.2, while Q2-Q4-Q6-Q8 scored 2.3 ± 0.2. These results indicate that the users agreed more with the positive sentences and disagreed more with negative ones. The NAU (range 1–7) showed high ratings of 5.6 ± 0.5, and the ASQ (range 1–5) showed an average value of 3.7 ± 0.8.

During the clinical trial, the psychophysical platform stored 1,913 measurements.

### Usage Metrics

The time passed between a measurement and the subsequent have been calculated and plotted in Figure 5. Records were aggregated by day and outliers due to technical problems and breaks for the subject were ignored, the mean time between two measurements is 74 ± 31.8 s. The number of measurements per day have also being considered an indicator of the overall use of the platform, the mean use overall was 128 ± 39.6 measurements per day. Each measurement consisted of a.mat file of roughly 1.14 kB, the whole dataset totaled then for almost 2.2 MB.

## Discussion

Electrical stimulation has been proposed as a way of restoring somatosensations (15, 63–67) in cases where they have been lost due to injury or disease in both the CNS or the PNS. In fact, sensory feedback is crucial to improve the motor control of robotic limbs or prostheses, enabling the patient to be more efficient in manipulating objects (4, 28, 55, 68). The sensations evoked thus had to be characterized in detail in patients receiving stimulation in order to restore the sensory information. The psychometric questionnaires were able to register all the aspects of the sensations being restored in a reliable and efficient way, considering more descriptors than in previous studies (34) and using a user-friendly platform.

Currently, there are many important sensation properties which need to be collected in order to obtain an intuitive and rich sensory feedback. In particular, the sensation location, type, quality, and intensity are valid and extendable for all the approaches in different neurological conditions. Considering the previously presented interface to collect stimulation-evoked somatosensory percepts, Geng et al. (35) showed a platform used to evaluate electrical stimulation to relieve PLP. Their platform was interfaceable with one type of neural stimulator and contained three questions to characterize the evoked sensation considering 12 sensation descriptors. The psychometric platform presented here reports somatosensory percepts based on five questionnaires containing 20 standard sensory descriptors (Table 1). The platform exploits a customizable, fast, and easy-to-use GUI which can be efficiently connected to several neural stimulators (31, 69, 70).

Since several groups are currently using electrical stimulation to restore sensory feedback, a standard somatosensory platform could facilitate their comparison, assessment, and optimization. Our findings support the conclusion that this psychometric platform could help and accelerate the development of sensorimotor neuroprostheses.

Given the simple software architecture, this platform is flexible in terms of modifications and upgrades. It is possible to add new questionnaires regarding other aspects of sensory feedback restoration. For example, two important features to be considered for the development of the next generation of somatosensory neuroprostheses are embodiment (42, 71, 72) and psychological/affective aspects (73).

The psychometric platform is simple to interface with other devices and also with existing software, thanks to its open and platform-agnostic interfaces: in ODF mode the HTTP interface accepts commands regardless of the device and the programming language of the sender application (all major languages can implement HTTP communication effortlessly). Answers to the questionnaires are exported in a CSV format, making it easy for any other software program to import and analyze them.

Considering the results of the usability assessments (Figure 5), users highlighted various positive and negative aspects which will then help us to improve the platform. The most positive aspect in terms of the “overall reaction to the software” was that the software is easy to use, which is crucial both for patients and experimenters.

The QUIS answers revealed that this system is consistent and very clear; however, we still need to improve error and warning messages. These aspects mainly regard the experimenters' side. The SUS again indicated that the system is easy to use and intuitive, but additional material and instructions should be included as support. Moreover, the NAU showed a high user satisfaction along with a request for more error messages. Finally, the ASQ revealed “the ease of completing this task,” thus highlighting the need for more support, information, and documentation. We thus intend to improve the platform using these usability results.

Usage metrics confirmed the ease of the fill-in process, with a mean time needed for a complete session of 74 s. Thanks to that stimulation spots were probed with a suitable rate (128 ± 39.6 measurements per day), for the intended objectives. The small dimension of the measurements made easy the subsequent steps of data revision, analysis, and storage, anyway authors are planning further reduction in the memory needed.

## Study Limitations

There are several limitations connected to the patient attention at the time of testing. To solve this issue, it is important to repeat the test multiple times over multiple days in order to increase its reliability. The test is also highly subjective, and the mapping results could strongly depend on the sensation of the patient and his/her personal experience (74). The individual subjective differences remain a big challenge for interpreting the somatosensory results and also the semantic differences. The usability and the utility of the platform need to be demonstrated with a larger population of patients with sensory deficits and with other technologies for the restoration of sensory feedback (e.g., non-invasive stimulation, brain stimulation).

Sham (placebo) and blind stimulations could also be delivered to test individual response bias and identify possible unreliable self-reports.

The software design, particularly the GUI, was inspired by the principles of the ISO 9241 standard. In fact, the users' opinions of the platform were taken into consideration during the design phase and the assessment.

The software will be actively used during experiments, and the user experience will be monitored to improve new versions, ensuring an iterative development driven by user feedback, as also stated in ISO 9241. In particular, in addition to the online availability of the software, the platform needs to be used in future clinical trials for both upper- and lower-limb amputees provided with a fully implantable sensory feedback system in long-term studies.

## Conclusions

This study has presented a psychometric platform used to record a complete somatosensory percept description, which can be evoked by several different methods of electrical stimulation in humans. The subjective somatosensory sensation type, location, quality, and intensity are collected and used to develop a somatosensory questionnaire, which can be used for neuroprosthesis calibration and optimization. The psychometric toolbox is implemented in a user-friendly software program. The platform was validated in patients with electrodes implanted in the PNS.

We believe that this new somatosensory psychometric system will help to establish a standard and uniform methodology of subjective sensory reports, which is a pivotal step to uniformly develop, adapt, and improve somatosensory neuroprostheses.

## Data Availability Statement

The datasets generated during and/or analyzed during the current study are available from the corresponding author on reasonable request. The Easy Quest Android application is available from the Google Play Store platform: https://play.google.com/store/apps/details?id=me.francescoiberite.research.easy_quest.

## Ethics Statement

The studies involving human participants were reviewed and approved by Institutional Ethics Committees of Policlinic A. Gemelli at the Catholic University and Italian Ministry of Health. The patients/participants provided their written informed consent to participate in this study. Written informed consent was obtained from the individual(s) for the publication of any potentially identifiable images or data included in this article.

## Author Contributions

GV designed the study, developed the software, analyzed the data, and wrote the paper. FI developed the software, analyzed the data, and reviewed the manuscript. IS designed the study, developed the software, and reviewed the manuscript. ED'A developed the software and reviewed the manuscript. GG, RD, and PR tested the platform with patients. TS developed the TIME electrodes. TS and PR discussed the results and reviewed the manuscript. SR designed the platform. FP designed the platform and discussed the results. SM designed the study, discussed the results, and reviewed the manuscript. All authors read, commented, and approved the manuscript.

## Conflict of Interest

FP, SR, and SM hold shares of Sensars Neuroprosthetics Sarl, a start-up company dealing with potential commercialization of neurocontrolled artificial limbs. The remaining authors declare that the research was conducted in the absence of any commercial or financial relationships that could be construed as a potential conflict of interest.

## Supplementary Material

Supplementary Video 1 Example of collection of somatosensory sensation data. The video shows the ODF procedure, where the Easy Quest app and the software controlling the stimulator interact. After the login procedure, the user sets the android application in ODF, opening the communication port. As soon as the controller software, after a verification procedure, detects the running app, there is a visual confirmation in the dashboard on the right-hand side of the screen. When stimulation parameters are correctly set, the software can deliver the stimulation and automatically send a sensation questionnaire to the subject's device. Instead of being stored in the device, the answers are sent back to the control software and are shown in the lower part of the interface.

## References

[1] Borton D Micera S Millan J del R Courtine G. Personalized neuroprosthetics. Sci Transl Med. (2013) 5:210rv2. 10.1126/scitranslmed.3005968 24197737

[2] Klaes C Shi Y Kellis S Minxha J Revechkis B Andersen RA. A cognitive neuroprosthetic that uses cortical stimulation for somatosensory feedback. J Neural Eng. (2014) 11:056024. 10.1088/1741-2560/11/5/056024 25242377 PMC4410973

[3] Ono T Shindo K Kawashima K Ota N Ito M Ota T. Brain-computer interface with somatosensory feedback improves functional recovery from severe hemiplegia due to chronic stroke. Front Neuroeng. (2014) 7:19. 10.3389/fneng.2014.00019 25071543 PMC4083225

[4] Raspopovic S Capogrosso M Petrini FM Bonizzato M Rigosa J Di Pino G. Restoring natural sensory feedback in real-time bidirectional hand prostheses. Sci Transl Med. (2014) 6:222ra19. 10.1126/scitranslmed.3006820 24500407

[5] Tan DW Schiefer MA Keith MW Anderson JR Tyler J Tyler DJ. A neural interface provides long-term stable natural touch perception. Sci Transl Med. (2014) 6:257ra138. 10.1126/scitranslmed.3008669 25298320 PMC5517305

[6] Flesher SN Collinger JL Foldes ST Weiss JM Downey JE Tyler-Kabara EC. Intracortical microstimulation of human somatosensory cortex. Sci Transl Med. (2016) 8:361ra141. 10.1126/scitranslmed.aaf8083 27738096

[7] Hiremath SV Tyler-Kabara EC Wheeler JJ Moran DW Gaunt RA Collinger JL. Human perception of electrical stimulation on the surface of somatosensory cortex. PLoS ONE. (2017) 12:e0176020. 10.1371/journal.pone.0176020 28489913 PMC5425101

[8] Salas MA Bashford L Kellis S Jafari M Jo H Kramer D. Proprioceptive and cutaneous sensations in humans elicited by intracortical microstimulation. eLife Sci. (2018) 7:e32904. 10.7554/eLife.32904 29633714 PMC5896877

[9] Valle G Mazzoni A Iberite F D'Anna E Strauss I Granata G. Biomimetic intraneural sensory feedback enhances sensation naturalness, tactile sensitivity, and manual dexterity in a bidirectional prosthesis. Neuron. (2018). 10.1016/j.neuron.2018.08.033 30244887

[10] Overstreet CK Cheng J Keefer E. Fascicle specific targeting for selective peripheral nerve stimulation. J. Neural Eng. (2019) 16:066040. 10.1088/1741-2552/ab4370 31509815

[11] Petrini FM Bumbasirevic M Valle G Ilic V Mijović P Cvančara P. Sensory feedback restoration in leg amputees improves walking speed, metabolic cost and phantom pain. Nat Med. (2019) 25:1356–63. 10.1038/s41591-019-0567-3 31501600

[12] Petrini FM Valle G Bumbasirevic M Barberi F Bortolotti D Cvancara P. Enhancing functional abilities and cognitive integration of the lower limb prosthesis. Sci Transl Med. (2019) 11:eaav8939. 10.1126/scitranslmed.aav8939 31578244

[13] Chandrasekaran S Nanivadekar AC McKernan G Helm ER Boninger ML Collinger JL. Sensory restoration by epidural stimulation of the lateral spinal cord in upper-limb amputees. eLife. (2020) 9:e54349. 10.7554/eLife.54349.sa2 32691733 PMC7373432

[14] Ortiz-Catalan M Mastinu E Sassu P Aszmann O Brånemark R. Self-contained neuromusculoskeletal arm prostheses. N Engl J Med. (2020) 382:1732–8. 10.1056/NEJMoa1917537 32348644

[15] Heming EA Choo R Davies JN Kiss ZHT. Designing a thalamic somatosensory neural prosthesis: consistency and persistence of percepts evoked by electrical stimulation. IEEE Trans Neural Syst Rehabil Eng. (2011) 19:477–82. 10.1109/TNSRE.2011.2152858 21622082

[16] Chai G Sui X Li S He L Lan N. Characterization of evoked tactile sensation in forearm amputees with transcutaneous electrical nerve stimulation. J Neural Eng. (2015) 12:066002. 10.1088/1741-2560/12/6/066002 26401550

[17] D'Anna E Petrini FM Artoni F Popovic I Simanić I Raspopovic S. A somatotopic bidirectional hand prosthesis with transcutaneous electrical nerve stimulation based sensory feedback. Sci Rep. (2017) 7:10930. 10.1038/s41598-017-11306-w 28883640 PMC5589952

[18] Osborn LE Dragomir A Betthauser JL Hunt CL Nguyen HH Kaliki RR. Prosthesis with neuromorphic multilayered e-dermis perceives touch and pain. Sci Robotics. (2018) 3:1–11. 10.1126/scirobotics.aat3818 32123782 PMC7051004

[19] Slopsema JP Boss JM Heyboer LA Tobias CM Draggoo BP Finn KE. Natural sensations evoked in distal extremities using surface electrical stimulation. Open Biomed Eng J. (2018) 12:1–15. 10.2174/1874120701812010001 29541258 PMC5842387

[20] Rothwell JC Traub MM Day BL Obeso JA Thomas PK Marsden CD. Manual motor performance in a deafferented man. Brain. (1982) 105:515–42. 10.1093/brain/105.3.515 6286035

[21] Jarrassé N Kühne M Roach N Hussain A Balasubramanian S Burdet E. Analysis of grasping strategies and function in hemiparetic patients using an instrumented object. In: 2013 IEEE 13th International Conference on Rehabilitation Robotics (ICORR). Seattle, WA (2013). p. 1–8. 10.1109/ICORR.2013.6650379 24187198

[22] Graczyk EL Schiefer MA Saal HP Delhaye BP Bensmaia SJ Tyler DJ. The neural basis of perceived intensity in natural and artificial touch. Sci Transl Med. (2016) 8:362ra142. 10.1126/scitranslmed.aaf5187 27797958 PMC5713478

[23] Valle G Petrini FM Strauss I Iberite F D'Anna E Granata G. Comparison of linear frequency and amplitude modulation for intraneural sensory feedback in bidirectional hand prostheses. Sci Rep. (2018) 8:16666. 10.1038/s41598-018-34910-w 30420739 PMC6232130

[24] Valle G Strauss I D'Anna E Granata G Di Iorio R Stieglitz T. Sensitivity to temporal parameters of intraneural tactile sensory feedback. J NeuroEngineering Rehabil. (2020) 17:110. 10.1186/s12984-020-00737-8 32799900 PMC7429895

[25] Raspopovic S Petrini FM Zelechowski M Valle G. Framework for the development of neuroprostheses: from basic understanding by sciatic and median nerves models to bionic legs and hands. Proc IEEE. (2017) 105:34–49. 10.1109/JPROC.2016.2600560

[26] Romeni S Valle G Mazzoni A Micera S. Tutorial: a computational framework for the design and optimization of peripheral neural interfaces. Nat Protoc. (2020) 15:3129–53. 10.1038/s41596-020-0377-6 32989306

[27] Zelechowski M Valle G Raspopovic S. A computational model to design neural interfaces for lower-limb sensory neuroprostheses. J NeuroEng Rehabil. (2020) 17:24. 10.1186/s12984-020-00657-7 32075654 PMC7029520

[28] Schiefer M Tan D Sidek SM Tyler DJ. Sensory feedback by peripheral nerve stimulation improves task performance in individuals with upper limb loss using a myoelectric prosthesis. J Neural Eng. (2016) 13:016001. 10.1088/1741-2560/13/1/016001 26643802 PMC5517302

[29] Clemente F Valle G Controzzi M Strauss I Iberite F Stieglitz T. Intraneural sensory feedback restores grip force control and motor coordination while using a prosthetic hand. J Neural Eng. (2019) 16:026034. 10.1088/1741-2552/ab059b 30736030

[30] Tan DW Schiefer MA Keith MW Anderson JR Tyler DJ. Stability and selectivity of a chronic, multi-contact cuff electrode for sensory stimulation in human amputees. J Neural Eng. (2015) 12:026002. 10.1088/1741-2560/12/2/026002 25627310 PMC5517311

[31] Petrini FM Valle G Strauss I Granata G Di Iorio R D'Anna E. Six-months assessment of a hand prosthesis with intraneural tactile feedback. Ann Neurol. (2018) 85:137–54. 10.1002/ana.25384 30474259

[32] George JA Page DM Davis TS Duncan CC Hutchinson DT Rieth LW. Long-term performance of Utah slanted electrode arrays and intramuscular electromyographic leads implanted chronically in human arm nerves and muscles. J Neural Eng. (2020) 17:056042. 10.1088/1741-2552/abc025 33045689

[33] Hughes CL Flesher SN Weiss JM Downey JE Collinger JL Gaunt RA. Neural stimulation and recording performance in human somatosensory cortex over 1500 days. medRxiv [preprint]. (2020). 10.1101/2020.01.21.20018341 PMC8500669 34320481

[34] Lenz FA Seike M Richardson RT Lin YC Baker FH Khoja I. Thermal and pain sensations evoked by microstimulation in the area of human ventrocaudal nucleus. J Neurophysiol. (1993) 70:200–12. 10.1152/jn.1993.70.1.200 8360716

[35] Geng B Harreby KR Kundu A Yoshida K Boretius T Stieglitz T. Developments towards a psychophysical testing platform - a computerized tool to control, deliver and evaluate electrical stimulation to relieve phantom limb pain. In: Dremstrup K Rees S Jensen ØM editors. 15th Nordic-Baltic Conference on Biomedical Engineering and Medical Physics (NBC 2011) IFMBE Proceedings. Berlin: Springer (2011). p. 137–40.

[36] Kim LH McLeod RS Kiss ZHT. A new psychometric questionnaire for reporting of somatosensory percepts. J Neural Eng. (2018) 15:013002. 10.1088/1741-2552/aa966a 29076455

[37] Wewers ME Lowe NK. A critical review of visual analogue scales in the measurement of clinical phenomena. Res Nurs Health. (1990) 13:227–36. 10.1002/nur.4770130405 2197679

[38] Bouhassira D Attal N Fermanian J Alchaar H Gautron M Masquelier E. Development and validation of the neuropathic pain symptom inventory. Pain. (2004) 108:248–57. 10.1016/j.pain.2003.12.024 15030944

[39] Boretius T Badia J Pascual-Font A Schuettler M Navarro X Yoshida K. A transverse intrafascicular multichannel electrode (TIME) to interface with the peripheral nerve. Biosens Bioelectr. (2010) 26:62–9. 10.1016/j.bios.2010.05.010 20627510

[40] Rossini PM Micera S Benvenuto A Carpaneto J Cavallo G Citi L. Double nerve intraneural interface implant on a human amputee for robotic hand control. Clin Neurophysiol. (2010) 121:777–83. 10.1016/j.clinph.2010.01.001 20110193

[41] Granata G Di Iorio R Romanello R Iodice F Raspopovic S Petrini F. Phantom somatosensory evoked potentials following selective intraneural electrical stimulation in two amputees. Clin Neurophysiol. (2018) 129:1117–20. 10.1016/j.clinph.2018.02.138 29625342

[42] Rognini G Petrini FM Raspopovic S Valle G Granata G Stauss I. Multisensory bionic limb to achieve prosthesis embodiment and reduce distorted phantom limb perceptions. J Neurol Neurosurg Psychiatry. (2018) 1–3. 10.1136/jnnp-2018-318570 30100550 PMC6791810

[43] Risso G Valle G Iberite F Strauss I Stieglitz T Controzzi M. Optimal integration of intraneural somatosensory feedback with visual information: a single-case study. Sci Rep. (2019) 9:7916. 10.1038/s41598-019-43815-1 31133637 PMC6536542

[44] Strauss I Valle G Artoni F D'Anna E Granata G Iorio RD. Characterization of multi-channel intraneural stimulation in transradial amputees. Sci Rep. (2019) 9:1–11. 10.1038/s41598-019-55591-z 31848384 PMC6917705

[45] Granata G Valle G Di Iorio R Iodice F Petrini FM Strauss I. Cortical plasticity after hand prostheses use: is the hypothesis of deafferented cortex “invasion” always true? Clin Neurophysiol. (2020) 131:2341–8. 10.1016/j.clinph.2020.06.023 32828036

[46] Mazzoni A Oddo CM Valle G Camboni D Strauss I Barbaro M. Morphological neural computation restores discrimination of naturalistic textures in trans-radial amputees. Sci Rep. (2020) 10:1–14. 10.1038/s41598-020-57454-4 31949245 PMC6965126

[47] Valle G D'Anna E Strauss I Clemente F Granata G Di Iorio R. Hand control with invasive feedback is not impaired by increased cognitive load. Front Bioeng Biotechnol. (2020) 8:287. 10.3389/fbioe.2020.00287 32318562 PMC7147827

[48] D'Anna E Valle G Mazzoni A Strauss I Iberite F Patton J. A closed-loop hand prosthesis with simultaneous intraneural tactile and position feedback. Sci Robot. (2019) 4:eaau8892. 10.1126/scirobotics.aau8892 33137741

[49] Dhillon GS Horch KW. Direct neural sensory feedback and control of a prosthetic arm. IEEE Trans Neural Syst Rehabil Eng. (2005) 13:468–72. 10.1109/TNSRE.2005.856072 16425828

[50] Davis TS Wark HAC Hutchinson DT Warren DJ O'Neill K Scheinblum T. Restoring motor control and sensory feedback in people with upper extremity amputations using arrays of 96 microelectrodes implanted in the median and ulnar nerves. J Neural Eng. (2016) 13:036001. 10.1088/1741-2560/13/3/036001 27001946

[51] Wendelken S Page DM Davis T Wark HAC Kluger DT Duncan C. Restoration of motor control and proprioceptive and cutaneous sensation in humans with prior upper-limb amputation via multiple Utah Slanted Electrode Arrays (USEAs) implanted in residual peripheral arm nerves. J Neuroeng Rehabil. (2017) 14:121. 10.1186/s12984-017-0320-4 29178940 PMC5702130

[52] Graczyk EL Resnik L Schiefer MA Schmitt MS Tyler DJ. Home use of a neural-connected sensory prosthesis provides the functional and psychosocial experience of having a hand again. Sci Rep. (2018) 8:9866. 10.1038/s41598-018-26952-x 29959334 PMC6026118

[53] George JA Kluger DT Davis TS Wendelken SM Okorokova EV He Q. Biomimetic sensory feedback through peripheral nerve stimulation improves dexterous use of a bionic hand. Sci Robot. (2019) 4:eaax2352. 10.1126/scirobotics.aax2352 33137773

[54] Zollo L Pino GD Ciancio AL Ranieri F Cordella F Gentile C. Restoring tactile sensations via neural interfaces for real-time force-and-slippage closed-loop control of bionic hands. Sci Robot. (2019) 4:eaau9924. 10.1126/scirobotics.aau9924 31620665 PMC6795534

[55] Mastinu E Engels LF Clemente F Dione M Sassu P Aszmann O. Neural feedback strategies to improve grasping coordination in neuromusculoskeletal prostheses. Sci Rep. (2020) 10:11793. 10.1038/s41598-020-67985-5 32678121 PMC7367346

[56] Tan D Tyler D Sweet J Miller J. Intensity modulation: a novel approach to percept control in spinal cord stimulation. Neuromodulation. (2016) 19:254–9. 10.1111/ner.12358 26479774

[57] Chin JP Diehl VA Norman KL. Development of an instrument measuring user satisfaction of the human-computer interface. In: Proceedings of the SIGCHI Conference on Human Factors in Computing Systems CHI'88. New York, NY: ACM (1988). p. 213–8. 10.1145/57167.57203

[58] Brooke J. SUS - A quick and dirty usability scale. Usability evaluation in industry. (1996). p. 189.

[59] Tullis TS Stetson JN. A comparison of questionnaires for assessing website usability. In: Usability professional association conference. (2004). p. 1–12.

[60] Usability Engineering: Book by Jakob Nielsen Nielsen Norman Group. (1994). Available online at: https://www.nngroup.com/books/usability-engineering/ (accessed September 13, 2018).

[61] Lewis JR. IBM computer usability satisfaction questionnaires: psychometric evaluation and Instructions for use. Int J Human Comput Interaction. (1995) 7:57–78. 10.1080/10447319509526110

[62] Graczyk EL Delhaye BP Schiefer MA Bensmaia SJ Tyler DJ. Sensory adaptation to electrical stimulation of the somatosensory nerves. J Neural Eng. (2018) 15:046002. 10.1088/1741-2552/aab790 29551756 PMC6034502

[63] Bensmaia SJ. Biological and bionic hands: natural neural coding and artificial perception. Philos Trans R Soc B Biol Sci. (2015) 370:20140209. 10.1098/rstb.2014.0209 26240424 PMC4528821

[64] Heming E Sanden A Kiss ZHT. Designing a somatosensory neural prosthesis: percepts evoked by different patterns of thalamic stimulation. J Neural Eng. (2010) 7:064001. 10.1088/1741-2560/7/6/064001 21084731

[65] Weber DJ Friesen R Miller LE. Interfacing the somatosensory system to restore touch and proprioception: essential considerations. J Motor Behav. (2012) 44:403–18. 10.1080/00222895.2012.735283 23237464

[66] Bensmaia SJ Miller LE. Restoring sensorimotor function through intracortical interfaces: progress and looming challenges. Nat Rev Neurosci. (2014) 15:313–25. 10.1038/nrn3724 24739786 PMC12278825

[67] Bensmaia SJ Tyler DJ Micera S. Restoration of sensory information via bionic hands. Nat Biomed Eng. (2020) 4:1–13. 10.1038/s41551-020-00630-8 33230305 PMC10233657

[68] Ortiz-Catalan M Hakansson B Branemark R. An osseointegrated human-machine gateway for long-term sensory feedback and motor control of artificial limbs. Sci Transl Med. (2014) 6:257re6. 10.1126/scitranslmed.3008933 25298322

[69] Andreu D Guiraud D Souquet G. A distributed architecture for activating the peripheral nervous system. J Neural Eng. (2009) 6:026001. 10.1088/1741-2560/6/2/026001 19213992

[70] Bisoni L Carboni C Raffo L Carta N Barbaro M. An HV-CMOS integrated circuit for neural stimulation in prosthetic applications. IEEE Trans Circuits Syst II Express Briefs. (2015) 62:184–8. 10.1109/TCSII.2014.2387679

[71] Giummarra MJ Georgiou-Karistianis Nellie Nicholls MER Gibson SJ Chou Bradshaw JL. Corporeal awareness and proprioceptive sense of the phantom. Br J Psychol. (2010) 101:791–808. 10.1348/000712610X492558 20346204

[72] Preatoni G Valle G Petrini FM Raspopovic S. Lightening the perceived weight of a prosthesis with cognitively integrated neural sensory feedback. Curr Biol. (2021) 31:1–7. 10.1016/j.cub.2020.11.069 33417885

[73] McGlone F Wessberg J Olausson H. Discriminative and affective touch: sensing and feeling. Neuron. (2014) 82:737–55. 10.1016/j.neuron.2014.05.001 24853935

[74] Meisenberg G Williams A. Are acquiescent and extreme response styles related to low intelligence and education? Personal Individual Differ. (2008) 44:1539–50. 10.1016/j.paid.2008.01.010

[75] Valle G Iberite F Strauss I D'Anna E Granata G Stieglitz T. A psychometric platform to collect somatosensory sensations for neuroprosthetic use. bioRxiv. (2020). 10.1101/2020.07.23.218222 PMC8757828 35047903

