# Corrigendum: A Psychometric Platform to Collect Somatosensory Sensations for Neuroprosthetic Use

**DOI:** 10.3389/fmedt.2022.866538

**Published:** 2022-02-24

**Authors:** Giacomo Valle, Francesco Iberite, Ivo Strauss, Edoardo D'Anna, Giuseppe Granata, Riccardo Di Iorio, Thomas Stieglitz, Stanisa Raspopovic, Francesco M. Petrini, Paolo M. Rossini, Silvestro Micera

**Affiliations:** ^1^The BioRobotics Institute, Scuola Superiore Sant'Anna, Pisa, Italy; ^2^Department of Health Sciences and Technology, Institute for Robotics and Intelligent Systems, ETH Zürich, Zurich, Switzerland; ^3^Bertarelli Foundation Chair in Translational Neuroengineering, Centre for Neuroprosthetics, School of Engineering, École Polytechnique Fédérale de Lausanne (EPFL), Institute of Bioengineering, Lausanne, Switzerland; ^4^Istituto di Ricovero e Cura a Carattere Scientifico (IRCCS)-Policlinic A. Gemelli Foundation, Institute of Neurology, Catholic University of the Sacred Heart, Rome, Italy; ^5^Laboratory for Biomedical Microtechnology, Department of Microsystems Engineering–IMTEK, Bernstein Center, BrainLinks-BrainTools Cluster of Excellence, University of Freiburg, Freiburg, Germany; ^6^Department Neuroscience and Neurorehabilitation, IRCCS S. Raffaele-Pisana, Rome, Italy

**Keywords:** neuroprosthesis, neurostimulation, electrodes, sensory feedback, amputees, psychophysics, somatosensations, platform

In the published article, there was an error regarding the affiliation for author “Paolo M. Rossini.” He was given affiliation 4 “Istituto di Ricovero e Cura a Carattere Scientifico (IRCCS)-Policlinic A. Gemelli Foundation, Institute of Neurology, Catholic University of the Sacred Heart, Rome, Italy” in error, instead his correction affiliation should read 6 “Department Neuroscience and Neurorehabilitation, IRCCS S. Raffaele-Pisana, Rome, Italy.”

The authors apologize for this error and state that this does not change the scientific conclusions of the article in any way. The original article has been updated.

## Publisher's Note

All claims expressed in this article are solely those of the authors and do not necessarily represent those of their affiliated organizations, or those of the publisher, the editors and the reviewers. Any product that may be evaluated in this article, or claim that may be made by its manufacturer, is not guaranteed or endorsed by the publisher.

